# NR4A1 Methylation Associated Multimodal Neuroimaging Patterns Impaired in Temporal Lobe Epilepsy

**DOI:** 10.3389/fnins.2020.00727

**Published:** 2020-07-14

**Authors:** Dongmei Zhi, Wenyue Wu, Bo Xiao, Shile Qi, Rongtao Jiang, Xingdong Yang, Jian Yang, Wenbiao Xiao, Chaorong Liu, Hongyu Long, Vince D. Calhoun, Lili Long, Jing Sui

**Affiliations:** ^1^Brainnetome Center and National Laboratory of Pattern Recognition, Institute of Automation, Chinese Academy of Sciences, Beijing, China; ^2^University of Chinese Academy of Sciences, Beijing, China; ^3^Department of Neurology, Xiangya Hospital, Central South University, Changsha, China; ^4^Department of Neurology, The Second Affiliated Hospital, Nanchang University, Nanchang, China; ^5^Tri-Institutional Center for Translational Research in Neuroimaging and Data Science (TReNDS), Georgia Institute of Technology, Georgia State University − Emory University, Atlanta, GA, United States; ^6^Department of Neurology, Beijing Haidian Hospital, Beijing, China; ^7^Beijing Engineering Research Center of Mixed Reality and Advanced Display, School of Optics and Electronics, Beijing Institute of Technology, Beijing, China; ^8^CAS Centre for Excellence in Brain Science and Intelligence Technology, Institute of Automation, Chinese Academy of Sciences, Beijing, China

**Keywords:** temporal lobe epilepsy, multimodal fusion, methylation levels of NR4A1, functional connectivity, fractional anisotropy, gray matter volume

## Abstract

DNA hypermethylation has been widely observed in temporal lobe epilepsy (TLE), in which NR4A1 knockdown has been reported to be able to alleviate seizure severity in mouse model, while the underlying methylation-imaging pathway modulated by aberrant methylation levels of NR4A1 remains to be clarified in patients with TLE. Here, using multi-site canonical correlation analysis with reference, methylation levels of NR4A1 in blood were used as priori to guide fusion of three MRI features: functional connectivity (FC), fractional anisotropy (FA), and gray matter volume (GMV) for 56 TLE patients and 65 healthy controls. Post-hoc correlations were further evaluated between the identified NR4A1-associated brain components and disease onset. Results suggested that higher NR4A1 methylation levels in TLE were related with impaired temporal-cerebellar and occipital-cerebellar FC strength, lower FA in cingulum (hippocampus), and reduced GMV in putamen, temporal pole, and cerebellum. Moreover, findings were also replicated well in both patient subsets with either right TLE or left TLE only. Particularly, right TLE patients showed poorer cognitive abilities and more severe brain impairment than left TLE patients, especially more reduced GMV in thalamus. In summary, this work revealed a potential imaging-methylation pathway modulated by higher NR4A1 methylation in TLE via data mining, which may impact the above-mentioned multimodal brain circuits and was also associated with earlier disease onset and more cognitive deficits.

## Introduction

Temporal lobe epilepsy (TLE) is the most common type of focal epilepsy, and seizures are often resistant to antiepileptic drugs (Labate et al., 2006). In contrast to other neuropsychiatric disorders such as autism, epilepsy has a relatively lower heritability estimated to be 32% (Chen et al., 2017) while an increasing number of studies have identified that epigenetics played a critical role in TLE (Kobow and Blümcke, 2014). DNA methylation is one of the best-known epigenetic modifications, which has been implicated in genome stability, and gene expression that affects brain development (Zulet and Iriarte, 2017). Numerous studies have observed abnormal DNA methylation in TLE patients, and more than 75% of DNA displayed predominant hypermethylation in TLE patients (Long et al., 2017).

While there are many potential genes of which methylation may play a pivotal role in the etiology of TLE, a previous study has suggested that nuclear receptor subfamily 4 group A member 1 (NR4A1) knockdown in mouse models can alleviate seizure severity and prolong onset latency of TLE (Zhang et al., 2016). NR4A1 has been implicated in mediating synaptic plasticity, acquiring neuroprotection and neuronal differentiation during central nervous system development in epilepsy (Zhang et al., 2009). Moreover, previous studies have identified that NR4A1 is an epilepsy-associated gene and its expression was up-regulated following seizure induction in both TLE patients and animal models (Watson and Milbrandt, 1989; Zhang et al., 2016). Seizure progression can also be attenuated by ameliorating DNA methylation (Kobow et al., 2013). All the above evidence demonstrates that methylation of NR4A1 may play an important role in epilepsy development and neuronal mechanisms.

Moreover, it has been found that NR4A1 was highly expressed in cortex, caudate, putamen, hippocampus, and cerebellum in the adult brain in mice and rats models (Zetterström et al., 1996). Numerous studies have also reported both functional and structural abnormalities in TLE patients (Doucet et al., 2013; Scanlon et al., 2013); for instance, it is consistently reported that TLE patients showed decreased fractional anisotropy (FA) in corpus callosum and temporal lobe (Slinger et al., 2016) reduced gray matter volume (GMV) in thalamus and cerebellum (Scanlon et al., 2013) and abnormal functional connectivity (FC) in the default-mode network (Zhang et al., 2010). Considering epigenetic abnormalities are responsible for both brain functional and structural reorganization, which may contribute to the formation of hyper-excitable circuits and seizure activity (Kobow and Blümcke, 2014) hence we hypothesized that aberrant NR4A1 methylation may participate in the formation of altered structural and functional brain networks, which may further lead to impaired cognitive and executive function in TLE patients.

In this work, using a goal-directed and supervised learning model called multi-site canonical correlation analysis with reference (MCCAR) (Qi et al., 2018a, b), we aim to explore the interrelationships between methylation levels of NR4A1 and three MRI features, including FC from functional MRI (fMRI), FA from diffusion MRI (dMRI), and GMV from structural MRI (sMRI). The supervised fusion model can simultaneously maximize the correlations of certain imaging components with the measure of interest, such as the methylation levels of NR4A1, and inter-modality covariation.

To the best of our knowledge, this is the first attempt to explore multimodal imaging-methylation covariation in epilepsy (56 TLE patients and 65 demographically matched healthy controls [HCs]) under the guidance of methylation levels of NR4A1. The same analysis was also performed on right TLE and left TLE subsets to compare the influence of lateral epileptic focus. *Post hoc* correlations between the identified NR4A1-associated multimodal components and clinical symptoms were further evaluated.

## Materials and Methods

### Participants

All subjects were collected from Xiangya Hospital, Central South University, including 56 TLE patients and 65 demographically matched HCs (Table 1). Patients underwent detailed clinical evaluation including seizure semiology collection, long-term video-EEG monitoring, high-resolution head MRI with epilepsy-specific protocol and neuropsychological testing. Clinical diagnosis of TLE was made independently by two certified neurologists in epilepsy based on the criteria defined by the International League Against Epilepsy (Scheffer et al., 2017). Seizure lateralization of each TLE patient was determined by assessing seizure semiology, epileptiform discharges on ictal and interictal EEG. Except for the presence of hippocampal sclerosis, all TLE patients demonstrated normal MRI, and none of the patients underwent surgical treatment. HCs had no history of alcohol/drug abuse, neurological/psychiatric illness, or MRI abnormalities. Written informed consent from each subject was obtained prior to participation in the study, and the study was approved by the Ethics Committee of Xiangya Hospital, Central South University.

**TABLE 1:** Demographic and clinical information of subjects.

	TLE (*N* = 56)	HC (*N* = 65)	*p*-Value
**Age, y**	31.0 ± 12.0	32.6 ± 11.5	0.45^*a*^
**Male/female**	21/35	35/30	0.10^*b*^
**Handedness, L/R**	0/56	0/65	−
**Epilepsy lateralization, L/B/R**	26/3/27	−	−
**Age of onset, y**	22.9 ± 13.0	−	−
**Hippocampus Sclerosis: yes/no**	8/48	0/65	−
**Epilepsy duration, y**	8.1 ± 6.3	−	−
**Seizure frequency (proportion)**			
< twice per month	22 (39.29%)	−	−
2−4 times per month	11 (19.64%)	−	−
>4 times per month	23 (41.07%)	−	−
**Number of AEDs (proportion)**		−	−
0	4 (7.14%)	−	−
1	26 (46.43%)	−	−
2	24 (42.86%)	−	−
3	2 (3.57%)	−	−
**MMSE**	27.4 ± 3.9	29.7 ± 0.8	2.61 × 10^–5*a*^
**HSCT**	11.0 ± 4.9	13.7 ± 3.2	1.30 × 10^–3*a*^
**Methylation of NR4A1**	0.34 ± 0.06	0.28 ± 0.06	8.47 × 10^–6*a*^

*Data are expressed as mean ± SD. ^a^Two-sample t-test; ^b^Chi-square test. TLE, temporal lobe epilepsy; HC, healthy controls; L/B/R, left/bilateral/right epilepsy lateralization; AEDs, Anti-Epileptic Drugs; MMSE, Minimum Mental State Examination; HSCT, Hayling Sentence Complete Test.*

### Neuropsychological Assessment

Neuropsychological assessments were carried out by two experienced neuropsychologists. The cognitive ability was measured with the Minimum Mental State Examination (MMSE) (Tombaugh and Mcintyre, 1992) which is one of the most commonly used scales in the evaluation of global cognitive status. The Hayling Sentence Complete Test (HSCT) which was designed to asses basic initiation speed and response suppression was also used to measure the executive function (Burgess and Shallice, 1996). In addition, duration of epilepsy and age of onset were also recorded for TLE patients.

### The Measure of Methylation Levels of NR4A1 in Peripheral Blood

Whole blood (3 ml) was collected from the peripheral vein in upper limb into an EDTA tube for 33 TLE patients and 40 HCs, and DNA were extracted according to the protocol in a commercial kit (Beijing Adly Biological Company, China). DNA concentration and purity were determined using a NanoDrop spectrophotometer. All samples had DNA concentration greater than 50 ng/ul and optic density ratio of maximal absorbent wavelengths at 260−280 nm (OD260/OD280) ranging from 1.60 to 2.10. DNA integrity and concentration were also confirmed by gel electrophoresis for further experimentations. Epigenome-wide DNA methylation profiling was performed via the Illumina Infinium Human Methylation 850K BeadChip (Illumina, Inc., United States) according to Illumina recommended protocols for the determination of methylation levels of 844,465 CpG sites. For each sample, 1 ug of genomic DNA was bisulfite-converted using the Zymo EZ DNA Methylation Kit, then went through amplification, fragmentation, precipitation, resuspension, and hybridization to 50-mer probes attached to Infinium Human Methylation 850K BeadArray. Unhybridized and non-specifically hybridized DNA was washed away, and chip underwent extension and staining. Signal was detected by iSCAN system and then converted by GenomeStudio analysis software. Methylation level at individual CpG was reported as a β-value, which ranges from 0 (unmethylated) to 1 (completely methylated). Differential methylation loci were detected by two-sample t test after FDR correction for 844,465 methylation loci. Importantly, after differentially methylated sites of NR4A1 were obtained, pyrosequencing was further performed in blood DNA samples from 10 TLE patients and 10 demographically matched HCs, as a measure of assay cross-validation.

### Feature Extraction and Correction

The image acquisition parameters and the preprocessing steps are presented in Supplementary “Imaging acquisition and preprocessing” section. Three representative MRI features (FC, FA, and GMV) were extracted and each modality was reshaped into a one-dimensional vector for each subject and stacked along with subjects, forming a matrix with dimension of No. subjects × [No. FC or No. voxels] (Qi et al., 2018a). Each feature matrices were further normalized across all voxels of all subjects to ensure all modalities contribute equally in fusion analysis. Age, gender, and mean FDs (only for FC) were all regressed out from FC, FA, and GMV, respectively, to reduce their potential impact on the neuroimaging data.

### Fusion With Reference

**FIGURE 1:**
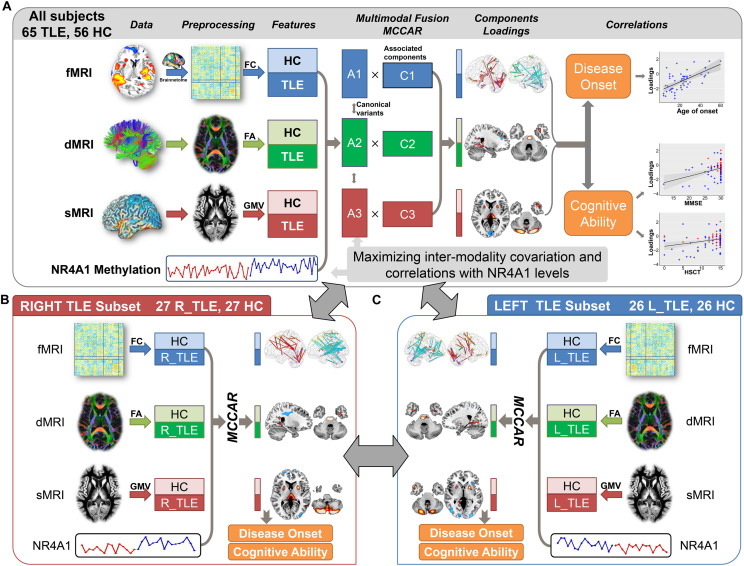
Flowchart of the NR4A1 methylation directed fusion analysis. **(A)** Methylation levels of NR4A1 were set as the reference to guide the three-way MRI fusion for all subjects. *Post hoc* correlation analysis was further conducted between the joint component and disease onset, and cognitive ability. The same fusion analysis was also performed on right TLE **(B)** and left TLE **(C)** subsets. Fusion results for left and right TLE subsets were further compared to investigate the influence of lateral epileptic focus.

NR4A1 methylation directed fusion analysis was implemented by feeding three preprocessing MRI features into MCCAR to explore the target joint components which are not only related with methylation levels of NR4A1, but also indicate significant functional–anatomical–structural alterations between TLE patients and HCs (Figure 1A). *Post hoc* correlations were further evaluated between loadings of the joint components and the disease onset, as well as cognitive abilities. In all fusion analysis, twenty components corresponding to the twenty canonical variants were determined for each modality using an advanced minimum description length criterion, and the regularization parameter λ was determined as 0.5 by a cross-validation method (Qi et al., 2018a). More detailed information about MCCAR was provided in Supplementary section “Multi-site canonical correlation analysis with reference,” and the effectiveness of this method, please refer to the study (Qi et al., 2018a).

Loading parameters of each component for each modality were compared using two-sample t-tests to identify the joint components, which showed significant group difference and correlations with methylation levels of NR4A1. We further performed a correlation analysis between loadings of joint components and clinical features, including MMSE scores, HSCT scores, age of onset and seizure duration. In order to explore the confounding effects of the medication on brain structure and functions, a correlation analysis was also conducted between the number of anti-epileptic drugs and loadings of joint components for TLE patients.

### Subset Replication

In order to verify the replicability of the findings, we performed the similar fusion analysis on different subsets. (1) In order to investigate the impact of lateral epileptic focus on the potential imaging-methylation pathway, the same NR4A1-directed fusion analysis was performed on left (*n* = 26) and right (*n* = 27) TLE patients, respectively, together with demographically matched HCs (Figures 1B,C). (2) The NR4A1-directed fusion analysis was also conducted on all subjects without hippocampal sclerosis to explore the effect of hippocampal sclerosis. (3) In addition, the NR4A1-directed fusion analysis was repeated 10 times on subgroups generated by randomly selecting two thirds of all participants from the whole samples.

## Results

### The Methylation Levels of NR4A1 in Peripheral Blood

DNA methylation assay showed that methylation levels of NR4A1 in TLE patients were significantly higher than those in HCs at one CpG site located in the promoter area, cg04416566 (TLE: 0.34 ± 0.06; HC: 0.28 ± 0.06; *p* = 0.019, *t* = 4.8, FDR corrected), and the group difference was still existed on right TLE and left TLE subgroups (Supplementary Figure S1). NR4A1 hypermethylation at cg04416566 site was further validated by pyrosequencing (TLE: 31.8 ± 1.5%; HC: 21.9 ± 3.2%; *p* = 4.91 × 10^–8^, *t* = 8.9), which were significantly correlated with the methylation levels of NR4A1 collected from Methylation 850K BeadChip (*r* = 0.92, *p* = 1.0 × 10^–8^).

### Multimodal Covarying Imaging Patterns Associated With Methylation Levels of NR4A1

**FIGURE 2:**
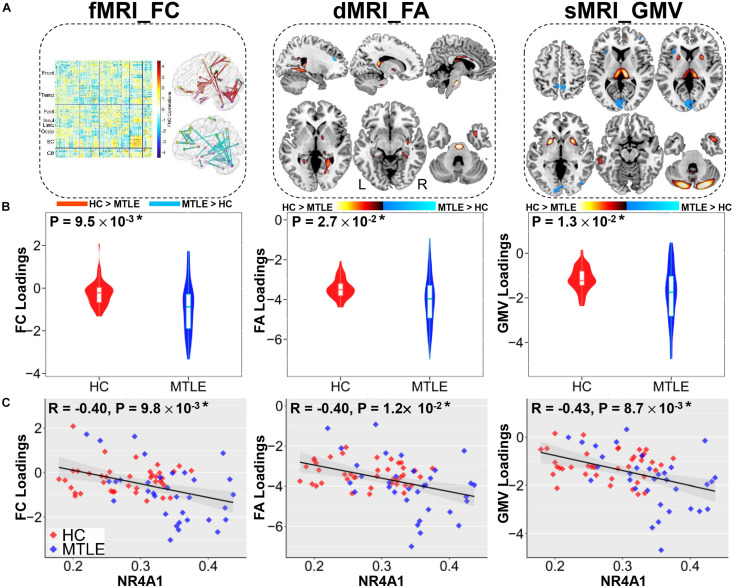
The joint components associated with methylation levels of NR4A1 show significant group difference in three modalities. **(A)** The spatial maps of FA and GMV were visualized at | Z| > 2, where the positive *Z*-values (red regions) denotes TLE < HC and the negative *Z*-values (blue regions) denotes TLE > HC. The FC matrix (left) was transformed into z-scores and visualized at | Z| > 3 (right), which displayed positive and negative links separately through the Brant toolbox (http://brant.brainnetome.org/en/latest/). **(B)** Group difference in loading parameters that were adjusted as HC > TLE on the mean of loadings for each modality. **(C)** Correlations between loadings of components and methylation levels of NR4A1 (HC, red dots; TLE, blue dots), in which TLE patients correspond to higher methylation levels of NR4A1 and higher loadings weights compared to HCs. Note that * means significance passed FDR corrected for multiple comparison (number of components * number of modalities) and gray regions in c indicate a 95% confidence interval.

Among twenty components, the first joint component was identified to be not only significantly group-discriminating (Two sample *t*-test: *p* = 9.5 × 10^–3*^, *t* = 3.8; *p* = 2.7 × 10^–2*^, *t* = 3.2; *p* = 1.3 × 10^–2*^, *t* = 3.7 for FC, FA, and GMV, respectively, ^∗^ denotes FDR corrected for multiple comparison) but also negatively correlated with methylation levels of NR4A1 (*r* = −0.40, −0.40, −0.43 for FC, FA, and GMV, respectively; *p* < 0.05^∗^ for all), indicating that lower loading parameters correspond to higher methylation levels of NR4A1 (Figure 2). Moreover, the joint components and the reference were also partially correlated after regressing out the group effect (*r* = −0.31, −0.32, −0.33 for FC, FA, and GMV, respectively; *p* < 0.01 for all). Other joint components were demonstrated in Supplementary Figure S2, and none of the components were significantly correlated with the methylation levels of NR4A1 except for the first joint component.

As displayed in Figure 2A, higher methylation levels of NR4A1 in TLE patients were found to be mainly related to both functional and structural alterations in subcortical nuclei, temporal network and cerebellum. For subcortical nuclei, increased methylation of NR4A1 in TLE patients were linked with decreased GMV in thalamus and putamen, lower FA in fornix (cres)/stria terminalis (FX/ST), internal capsule, and external capsule, together with reduced FC among thalamus, putamen, and globus pallidus. For temporal network, higher methylation levels of NR4A1 in TLE patients were associated with decreased GMV in inferior and middle temporal gyrus (ITG, MTG) and temporal pole, reduced FA in cingulum (hippocampus), inferior longitudinal fasciculus (ILF), and uncinate fasciculus (UF), as well as impaired FC within temporal lobe, and between temporal lobe and parietal lobe, and cerebellum. For cerebellum, results showed that higher methylation levels of NR4A1 were related to lower GMV in cerebellum, reduced FA in middle cerebellar peduncle (MCP), altered FC within cerebellum, and between cerebellum and temporal lobe, parietal lobe, and occipital lobe in TLE patients.

### Association With Clinical Measures

**FIGURE 3:**
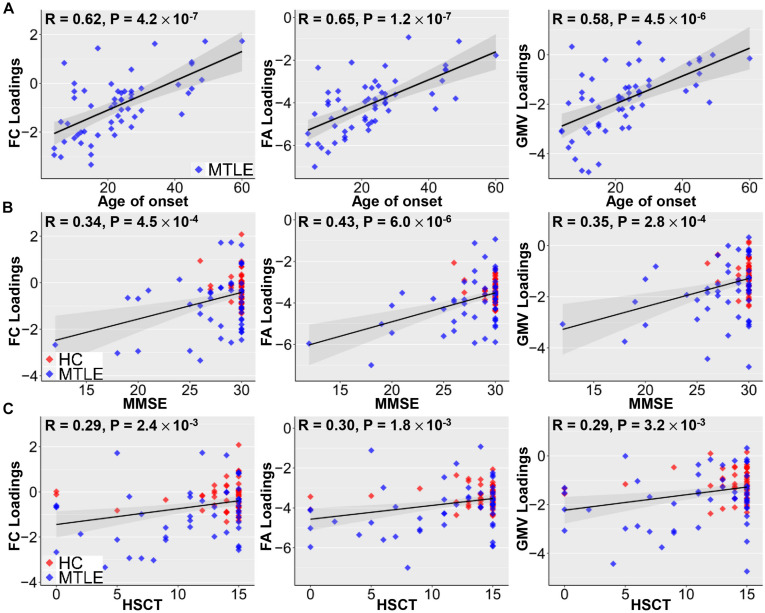
Correlations between loadings of identified components and clinical measures. Positive correlations between loadings of identified components and age of onset **(A)**, MMSE **(B)**, and HSCT scores **(C)**. Earlier TLE onset corresponds to lower loading weights in three modalities, suggesting more serious disability in epilepsy. Higher MMSE scores are associated with better cognitive ability and higher HSCT scores correspond to better executive function, which are linked with higher loading weights. Note that gray regions in panels **(A–C)** indicate a 95% confidence interval.

The identified joint ICs associated with methylation levels of NR4A1 were not only positively correlated with age of onset in TLE patients (FC: *r* = 0.62; FA: *r* = 0.65; GMV: *r* = 0.58; *p* < 1.0 × 10^–5^ for all, Figure 3A), but also related to poorer cognitive ability measured by MMSE (FC: *r* = 0.34; FA: *r* = 0.43; GMV: *r* = 0.35; *p* < 0.001 for all, Figure 3B), and worse executive function reflected by the accuracy of HSCT (FC: *r* = 0.29; FA: *r* = 0.30; GMV: *r* = 0.29; *p* < 0.005 for all, Figure 3C). No significant association was detected between the number of anti-epileptic drugs and loadings of joint components for TLE patients (FC: *r* = 0.06, *p* = 0.68; FA: *r* = −0.07, *p* = 0.60; GMV: *r* = −0.17; *p* = 0.21).

### NR4A1 Methylation Directed Fusion Analysis on Left and Right TLE Patients

**FIGURE 4:**
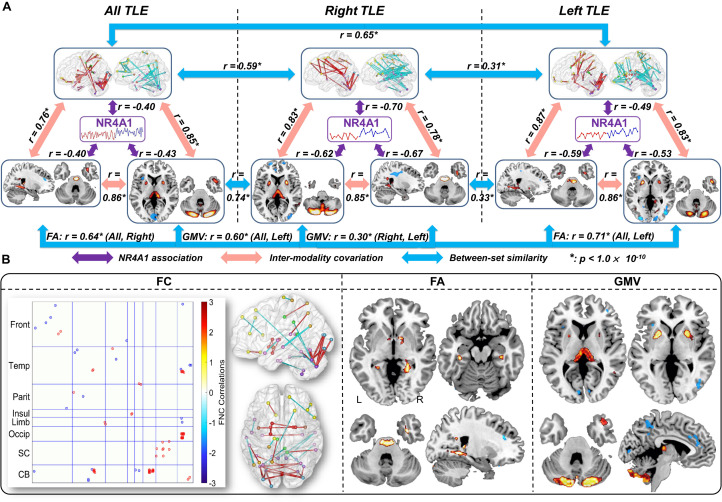
Comparison of the identified multimodal covarying patterns among all TLE, right TLE, and left TLE patients. **(A)** Spatial similarities among all TLE, right TLE, and left TLE patients. The purple arrows denote correlations between methylation of NR4A1 and the first joint components; the red arrows denote inter-modality covariation between different modalities in each subset analysis; and the blue arrows denote intra-modality similarities among three (sub) groups. * means *p* < 1.0 × 10^– 10^. **(B)** Overlap of spatial maps of FC, FA, and GMV among all, right, and left TLE patients.

The same NR4A1 methylation directed fusion analysis was performed on right TLE (Supplementary Figure S5) and left TLE subsets (Supplementary Figure S6) to investigate the influence of epileptic focus. Multimodal covarying patterns associated with methylation levels of NR4A1 were compared among all TLE, right TLE, and left TLE groups (Figure 4A). While significantly higher spatial similarities were demonstrated between all TLE and right TLE patients (*r* = 0.59, 0.64, 0.74 for FC, FA, and GMV, respectively, *p* < 1 × 10^–10^ for all), and left TLE patients (*r* = 0.65, 0.71, 0.60 for FC, FA, and GMV, respectively, *p* < 1 × 10^–10^ for all), lower spatial similarities were observed between right TLE and left TLE patients with *r* = 0.31, 0.33, 0.30 (*p* < 1 × 10^–10^) for FC, FA, and GMV, respectively. In addition, the joint components associated with methylation levels of NR4A1 were also pair-wisely correlated between modalities, indicating significant multimodal covariation across subjects.

Furthermore, we compared spatial overlap among these three groups (Figure 4B), in which components in any two or three groups are considered as overlapping components. Results revealed that all TLE, right TLE and left TLE patients were commonly impaired in thalamus, putamen, temporal pole, and cerebellum. Compared with left TLE, right TLE showed more significant group difference in the joint components, especially more GMV reduction in thalamus (Supplementary Figure S9). In addition, while NR4A1-associated multimodal components in left TLE were only associated with age of onset (Supplementary Figure S7), covarying components in right TLE were associated with age of onset, MMSE, and HSCT scores (Supplementary Figure S8), indicating more impaired cognitive and executive function in right TLE. Though no significant lateralization preference was observed in right TLE and left TLE, thalamus and putamen showed more impairment in GMV on the side of seizure origin compared with the contralateral side (Supplementary Figure S9).

### Subset Replication

The above findings were also replicated in 10 subgroup analyses (Supplementary Figure S3) and subjects without hippocampal sclerosis (Supplementary Figure S4), especially the brain impairment in putamen, thalamus, temporal pole, and cerebellum in TLE.

## Discussion

To the best of our knowledge, this is the first attempt to link the methylation of NR4A1 with multimodal brain imaging to investigate the imaging-methylation covariance via a goal-directed model in TLE. Results indicated that higher methylation of NR4A1 in TLE was related to covarying functional and structural impairment, including subcortical nuclei, temporal network and cerebellum, which were further related to earlier age of onset, impaired cognitive ability and executive function.

### Multimodal Covarying Imaging Patterns Associated With Methylation of NR4A1

Compared with HCs, TLE patients showed higher methylation of NR4A1, which is replicated in both right and left TLE patients, indicating that higher methylation of NR4A1 is correlated with TLE independent of seizure origin. According to previous studies, the occurrence of epilepsy is closely related to aberrant DNA methylation, which can further control its gene expression (Kobow et al., 2013; Dȩbski et al., 2016). The methylation of NR4A1 can be catalyzed by DNA methyltransferase enzymes, which are upregulated in TLE (Zhu et al., 2012). A recent study reported that abnormal DNA methylation together with other epigenetic changes can lead to diverse pathologies including altered neurogenesis, abnormal neuronal migration, and aberrant structure of individual cells, and/or large networks in epilepsy (Kobow and Blümcke, 2014), consistent with our results that increased methylation of NR4A1 in TLE patients was associated with impaired brain function and structure in subcortical nuclei, temporal network and cerebellum.

As shown in Figure 1, higher methylation of NR4A1 in TLE patients was associated with reduced GMV in thalamus and putamen, which were also identified in TLE patients in previous studies (Dreifuss et al., 2001; Pulsipher et al., 2007). The thalamus and putamen play a pivotal role in regulating cortical excitability and seizure propagation after the principal epileptogenic focus has become active (Dreifuss et al., 2001), which may serve as a physiologic synchronizer of seizures (Natsume et al., 2003). Reduced FA in internal capsule, together with decreased FC among thalamus, putamen, and globus pallidus, were also found in TLE patients. Thalamus receives information from medial temporal lobe including hippocampus and entorhinal cortex via fornix that was reduced in FA in TLE patients, which is involved in limbic circuit (Saunders et al., 2005). All the above results revealed that increased methylation of NR4A1 may affect both function and structure of subcortical nuclei in TLE patients.

Higher methylation of NR4A1 in TLE was associated with abnormal FC, FA, and GMV in most regions of the temporal lobe, consistent with the point that TLE originates in the temporal lobe and then spread to other cortex (Yoo et al., 2014). For instance, results showed temporal pole atrophy in GMV, which was related to age of epilepsy onset, indicating its role in the genesis and propagation of TLE (Coste et al., 2002; Abel et al., 2018). Reduced FA in ILF and UF that are connected to temporal pole were also found in TLE, which have been reported to be particularly vulnerable to pathological alterations (Kreilkamp et al., 2017). More interestingly, temporal polar hypometabolism has been found to be the most significant predicting factor of a good surgical outcome among other metabolic abnormalities detected by PET in TLE patients (Dupont et al., 2000). In addition, reduced FA-values in cingulum (hippocampus) suggested impaired limbic system in TLE patients, which is related to the length and severity of seizure activity (Chan et al., 1997). TLE patients also demonstrated reduced GMV in ITG and MTG, increased FC between MTG and IPL, and decreased FC between FG and cerebellum, suggesting widely impaired temporal regions in TLE patients. Furthermore, previous studies have identified that NR4A1 is an activity-inducible gene that regulates the density and distribution of spines and synapses which is related to excitatory synaptic strength (Chen et al., 2014), and mediates synaptic activity and neuroprotection in epilepsy, especially in temporal lobe (Zhang et al., 2009).

Higher methylation of NR4A1 was also related to reduced GMV in cerebellum and lower FA in MCP, consistent with cerebellar atrophy found in TLE in numerous studies (Hermann et al., 2005; Mcdonald et al., 2008). Reduced white matter integrity in cerebellum is implicated in impaired executive function in TLE patients (Riley et al., 2010), and the cerebellar-directed intervention has been proved to inhibit spontaneous temporal lobe seizures during the chronic phase of the disorder (Wong and Escayg, 2015). In addition, NR4A1 is implicated in differentiation into granule neuron that is the main type of neuron of cerebellum (Farmer, 2016). Therefore, increased methylation of NR4A1 may play a potential role in the impaired structure of cerebellum in TLE.

Multimodal covarying patterns associated with methylation levels of NR4A1 in right TLE and left TLE were remarkably consistent with that in all TLE. Compared with left TLE, right TLE showed more significant group difference, in line with previous results that right TLE patients exhibit more extensive impairment (Kim et al., 2016). For the identified joint components, while left TLE patients only show little impairment in left thalamus, right TLE patients showed bilateral impairment in thalamus, whose right thalamus activation is higher than left thalamus activation. Previous study found that asymmetric thalamic activation can predict the laterality of epileptic focus (Sojkova et al., 2003). The thalamus plays a critical role in the propagation of seizure activity to the cortical regions, which may be more prone to the neurotoxic effects of epileptiform discharges (Bonilha et al., 2005), especially in the ipsilateral side of epileptic focus.

### Relevance of Methylation of NR4A1 With Clinical Information

The identified covarying patterns associated with methylation levels of NR4A1 were correlated with age of onset in TLE patients. Consistent with our findings, Keller et al. (2002) have reported that decreased gray matter concentration in the thalamus and cerebellum are related to age of onset in TLE patients, and Leiva-Salinas et al. (2017) found that earlier seizure onset is correlated with the degree of temporal hypometabolism in TLE patients. More interestingly, it has been proved that NR4A1 knockdown by lentivirus transfection can prolong onset latency of epilepsy in mouse models (Zhang et al., 2016), implicating that methylation of NR4A1 may play a pivotal role in seizure onset. In addition, covarying patterns associated with higher methylation of NR4A1 were related to impaired cognitive and executive functions as measured by MMSE and HSCT test in TLE patients, which is in line with results that abnormal expression of NR4A1 lead to cognitive deficits, especially long-term memory (Bridi and Abel, 2013). All above results indicated that increased methylation of NR4A1 may lead to earlier seizure onset and poorer cognitive and executive function in TLE patients.

One possible limitation of this work is that we only choose the methylation of one gene which has been implicated in suppressing seizure activity in TLE patients (Zhang et al., 2016), i.e., NR4A1, while there may exist many other target genes related to TLE deserving further investigation. In addition, methylation levels and clinical information are unavailable for some subjects. In future, a larger number of subjects and more complete clinical information are preferred for the similar imaging-epigenetic association study. Finally, while we have investigated only correlations between multimodal brain imaging and methylation data, the intrinsic causality remains to be explored in more dedicated investigation on the pathology of TLE.

In summary, to the best of our knowledge, this is the first attempt to link the NR4A1 methylation levels with multimodal neuroimaging data in epilepsy, which were also replicated in both left and right TLE subsets. Though no significant lateralization preference was observed in right and left TLE patients, thalamus and putamen showed more impairment on the side of seizure origin compared with the contralateral side. Using a data-driven, supervised fusion approach, we discovered that higher NR4A1 levels in TLE patients may be related to functional and structural impairment in thalamus, putamen, temporal pole, and cerebellum, which were further linked with earlier age of disease onset, and poorer executive function in epilepsy patients. These results implicated a potential imaging-methylation pathway modulated by higher NR4A1 methylation in TLE patients, which awaits for further replication in future studies.

## Data Availability Statement

The original contributions presented in the study are included in the article/Supplementary Material, further inquiries can be directed to the corresponding author/s.

## Ethics Statement

The studies involving human participants were reviewed and approved by Ethics Committee of the Xiangya Hospital, Central South University. The patients/participants provided their written informed consent to participate in this study.

## Author Contributions

JS and DZ designed the study. DZ and WW conducted the data analysis. DZ, WW, SQ, RJ, XY, JY, LL, VC, and JS wrote the manuscript. WW, BX, WX, CL, HL, and LL collected the data. All authors contributed to the article and approved the submitted version.

## Conflict of Interest

The authors declare that the research was conducted in the absence of any commercial or financial relationships that could be construed as a potential conflict of interest.
