# Supplementary material for: NR4A1 Methylation Associated Multimodal Neuroimaging Patterns Impaired in Temporal Lobe Epilepsy
Source: Front Neurosci. 2020 Jul 14;14:727. doi: 10.3389/fnins.2020.00727 (PMC7372187; doi:10.3389/fnins.2020.00727)
Supplement: Supplementary file 1 [file Data_Sheet_1.pdf]

Supplementary Material

1. Imaging acquisition and preprocessing

FMRI, dMRI, and sMRI for all subjects were acquired on a 3T General Electric (Signa HDx, USA) scanner with a 32-channel phased-array head coil. All subjects were instructed to keep their eye closed and stay awake during scanning. For fMRI, a total of 360 volumes of echo planar images were collected for each subject with parameters: repetition time (TR)/echo time (TE) = 2000/30 ms; matrix size = 64 × 64; field of view (FOV) = 220 × 220 mm^2; voxel size = 3.4375 × 3.4375 × 4.6; flip angle = 90°; 32 sequential ascending axial slices of 4 mm thickness.

DMRI was acquired with parameters: TR/TE = 12000/76.9 ms; matrix size = 128 × 128; FOV = 256 × 256 mm^2; slice thickness = 3 mm; number of slices = 55 (with no gap); voxel size = 2 × 2 × 3 mm^3; flip angle = 90°, and a total of 35 images were collected, including 3 non-diffusion-weighted image (b = 0 s/mm^2) and 32 diffusion-weighted images (b = 1000 s/mm^2) with 32 non-collinear gradient directions.

SMRI was collected by a magnetization-prepared rapid gradient echo sequence with parameters: TR/TE = 7.78/2.984 ms; flip angle = 7°; matrix size = 256 × 256; FOV = 256 × 256 mm^2; voxel size = 1 × 1× 1 mm^3; slices thickness = 1 mm; number of slices = 188.

The fMRI data were preprocessed using an automated analysis pipeline developed at the Brainnetome center (http://www.brainnetome.org/) based on the SPM8 (http://www.fil.ion.ucl.ac.uk/spm/) software according to the previous study (Du et al., 2018;Xu et al., 2018) , including the removal of the first 10 volumes, slice timing, motion correction, normalization into MNI space and resample into 3 × 3 × 3 mm^3 voxels, detrending and band-pass filtering (0.01Hz-0.08Hz), and spatially smoothing with a 6 mm full width half max (FWHM) Gaussian kernel. Six head motion parameters, white matter (WM) signal, cerebrospinal fluid signal (CSF), and global mean signal were set as nuisance covariates to regressed out from fMRI data. We also excluded the subjects with a maximum translation of > 3 mm, rotation of > 3°, or mean framewise displacements (mean FD) > 0.5 mm to minimize the impact of head motion, and there is no significant group difference between TLE patients and HCs on mean FDs (TLE: 0.14 ± 0.08; HC: 0.12 ± 0.06; p = 0.09, t = 1.70). FC matrices were calculated between whole ROI time courses for each subject using Pearson correlation based on 274 ROIs (without 255) from Brainnetome Atlas (http://atlas.brainnetome.org/bnatlas.html). FC values were Fisher-Z transformed for subsequent analyses.

DMRI data were preprocessed using FMRIB Software Library (FSL; http://www.fmrib.ox.ac.uk/fsl). Any gradient direction with excessive motion or vibration artifacts was identified and removed. Eddy current distortions and head motion were corrected by aligning all diffusion-weighted images to the non-diffusion-weighted image, and gradient directions were corrected for any image rotation during the motion correction. Then the diffusion tensor and scalar measures such as FA were estimated and smoothed with a 6 mm FWHM Gaussian filter.

SMRI data were segmented into WM, grey matter, and CSF with non-linearly modulated normalized parameters via the unified segmentation method using VBM8 toolbox in SPM8 software package. The whole-brain grey matter volume (GMV) were smoothed with a 6 mm FWHM Gaussian

filter.

2. Group difference of methylation levels of NR4A1

Methylation levels of NR4A1 in different groups were compared. Results showed that TLE patients exhibited higher methylation levels of NR4A1 compared to HCs (TLE: 0.34 ± 0.06; HC: 0.28 ± 0.06; p = 0.019, t = 4.8, FDR corrected). Such a group difference still existed between right TLE patients and matched HCs ($p = 1.05 \times 10^{-4}$, t = 4.5, uncorrected), and between left TLE patients and matched HCs ($p = 8.50 \times 10^{-3}$, t = 2.8, uncorrected). In addition, there was no group difference in methylation levels of NR4A1 between left TLE and right TLE patients (p = 0.85, t = 0.20).

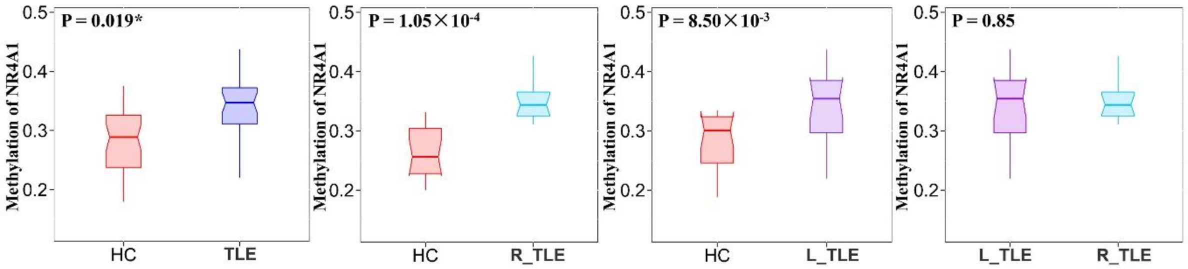

Figure S1. Comparison of methylation levels of NR4A1 between different groups. Note that * means significance passed FDR corrected for all methylation loci.

3. Multi-site canonical correlation analysis with reference

Multi-site canonical correlation analysis with reference (MCCAR) was proposed to simultaneously maximize the correlations of certain imaging components with the measure of interest, and inter-modality covariation. Assume X_k represents multimodal dataset and each is a linear mixture of components C_k with a nonsingular mixing matrix A_k, $k = 1, 2, 3$, denoting the modality. Namely, $X_k = A_k C_k$, where X_k is a feature matrix (subjects×voxels) and A_k is a mixing matrix (subjects×number of components). MCCAR imposes an additional constraint to maximize both the covariations among loadings of each modality, and the column-wise correlations between A_k and the reference signal, as shown in below equation.

$$\max \sum_{k,j=1}^{3} \left\{ \left\| corr(A_k, A_j) \right\|_2^2 + 2\lambda \cdot \left\| corr(A_k, ref) \right\|_2^2 \right\}$$

where ref is an $N \times 1$ vector, denoting the referred measure, N is the subject number. $corr(A_k, A_j)$ is the column-wise correlation between A_k and A_j, and $corr(A_k, ref)$ is the column-wise correlation between A_k and ref. After optimization by MCCAR, we can obtain the potential target components C_i that are correlated with ref in each modality, as well as being most correlated across subjects between modalities.

4. The identified 20 joint components

The first joint components were shown in **Figure 2** and the remaining nineteen components were shown in **Figure S2**. Among nineteen components, there were no significant group difference except for the fifth components (Two sample t-test, FC: p = 0.017, no significant group difference for FA and GMV features), and the fifteenth components (Two sample t-test, FC: p = 0.028, no significant for FA and GMV features), and none of the components was significantly correlated with the methylation levels of NR4A1.

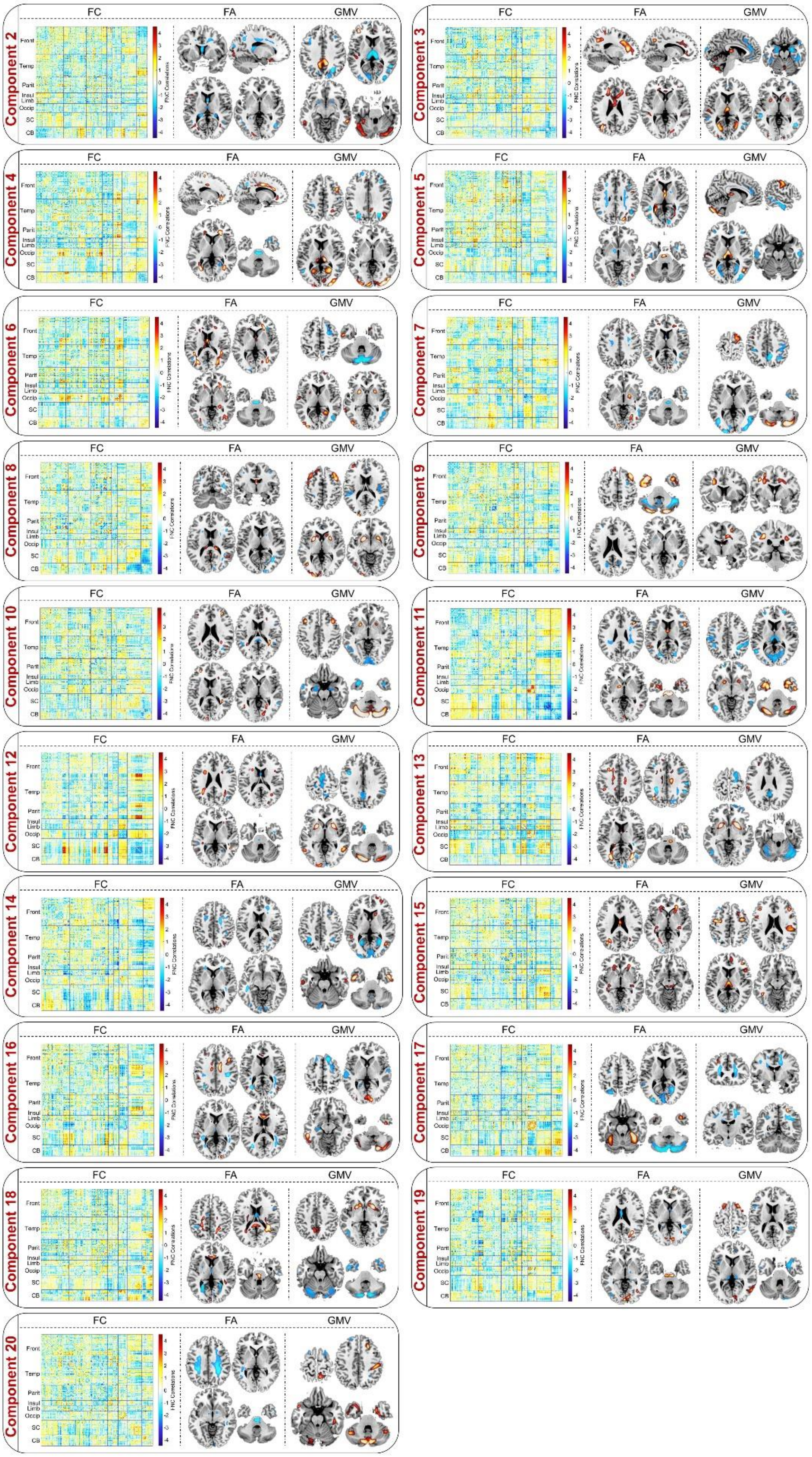

Figure S2. The identified twenty components except for the first joint components.

5. Subgroup validation analysis

The same NR4A1 methylation directed fusion analysis was repeated ten times on different subgroups to verify the replicability of the findings. The subgroup was generated by randomly selecting two thirds of all participants from the whole samples. In ten subgroup analyses, the first covarying components were identified to be both significantly group-discriminating (FC: $t_{mean} = 3.96 \pm 0.44$; FA: $t_{mean} = 3.44 \pm 0.53$; GMV: $t_{mean} = 3.70 \pm 0.46$; $p < 0.005$ for all modalities, uncorrected) and significantly correlated with methylation levels of NR4A1 (FC: $R_{mean} = -0.54 \pm 0.07$; FA: $R_{mean} = -0.55 \pm 0.06$; GMV: $R_{mean} = -0.53 \pm 0.08$; $p < 0.005$ for all modalities, uncorrected). Spatial overlap was further computed among ten subgroup analyses, in which FNC was thresholded at $|Z| > 3$, FA and GMV were thresholded at $|Z| > 2$. Results suggested that the commonly identified regions were the thalamus, putamen, temporal pole, and cerebellum (**Figure S3**).

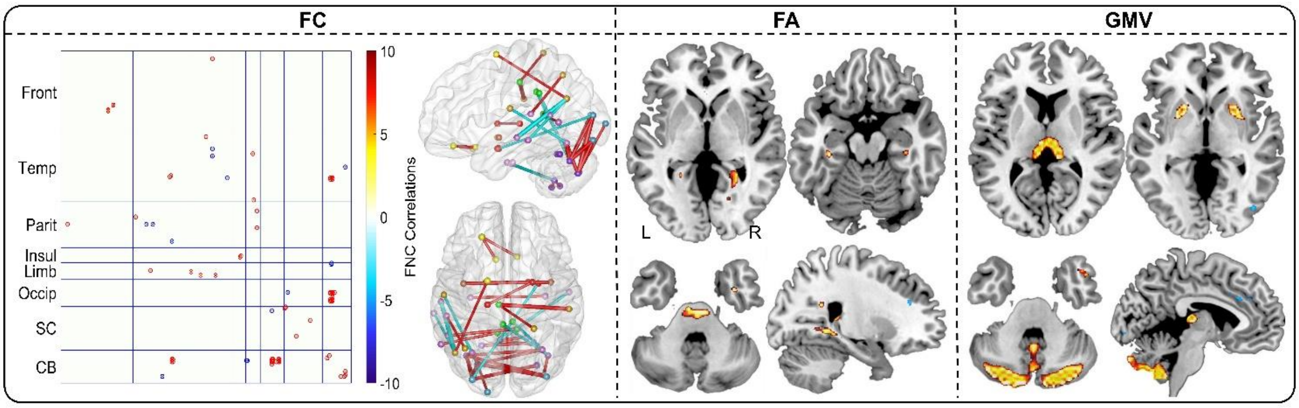

Figure S3. Spatial overlap among ten subsets validation analysis.

6. NR4A1-directed fusion analysis on subjects without hippocampal sclerosis

The NR4A1-directed fusion analysis was also conducted on all subjects without hippocampal sclerosis to investigate the effect of hippocampal sclerosis. Among twenty components, the first joint component was identified to be not only significantly group-discriminating (Two sample t-test: $p = 3.1 \times 10^{-3}$*, $t = 4.3$; $p = 3.5 \times 10^{-2}$*, $t = 3.3$; $p = 2.8 \times 10^{-3}$*, $t = 4.2$ for FC, FA, and GMV respectively, * denotes FDR corrected for multiple comparison) but also negatively correlated with methylation levels of NR4A1 (r = -0.50, -0.49, -0.48 for FC, FA, and GMV respectively; $p < 0.001$* for all three modalities), indicating that lower loading parameters correspond to higher methylation levels of NR4A1 (**Figure S4**). Correlation analyses were performed between the joint components and age of onset, MMSE, and HSCT scores, as shown in Table S1. The fusion results were consistent with all subjects, which may be due to small number of patients with hippocampal sclerosis. Future studies should include more patients with hippocampal sclerosis to investigate the influence of hippocampal sclerosis.

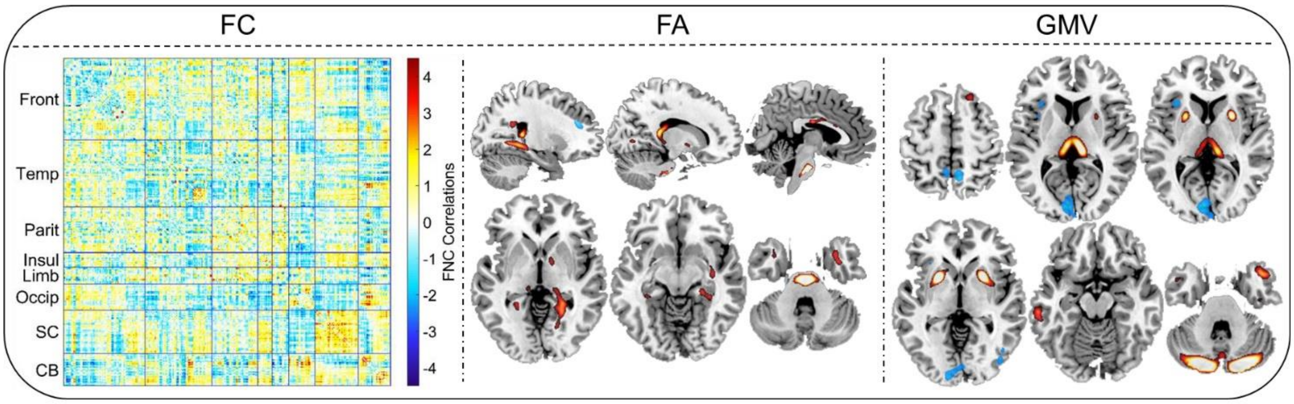

Figure S4. The first joint components associated with methylation levels of NR4A1 on subjects without hippocampal sclerosis in three modalities. The spatial maps of FA and GMV were visualized at |Z| > 2, where the positive Z-values (red regions) denotes TLE < HC and the negative Z-values (blue regions) denotes TLE > HC. The FC matrix (left) was transformed into z-scores and visualized at |Z| > 3 (right), which displayed positive and negative links separately through the Brant toolbox.

Table S1. Correlations between the identified components and clinical measures

	**Age of onset**		**MMSE**		**HSCT**	
	R	P	R	P	R	P
**Loadings of FC**	0.64	8.3×10^{-7}	0.32	1.4×10^{-3}	0.29	3.9×10^{-3}
**Loadings of FA**	0.68	1.2×10^{-7}	0.42	2.1×10^{-5}	0.30	2.7×10^{-3}
**Loadings of GMV**	0.53	9.0×10^{-5}	0.37	1.9×10^{-4}	0.32	1.6×10^{-3}

7. NR4A1 methylation directed fusion analysis on right and left TLE subsets

Table S2. Demographic and clinical information of right TLE patients and matched HCs.

	Right TLE (N=27)	HC (N=27)	*p* Value
**Age, y**	30.1 ± 13.0	32.1 ± 9.7	0.51[a]
**Male/female**	12/15	17/10	0.28[b]
**Handedness, L/R**	0/27	0/27	—
**Epilepsy lateralization, L/B/R**	0/0/27	—	—
**Age of onset, y**	23.0 ± 14.7	—	—
**Hippocampus Sclerosis: yes/no**	6/21	0/27	—
**Epilepsy duration, y**	7.0 ± 5.2	—	—
**Seizure frequency (proportion)**			
< twice per month	9 (33.33%)	—	—
2-4 times per month	7 (25.93%)	—	—
>4 times per month	11 (40.74%)	—	—
**Number of AEDs（proportion）**		—	—
0	1 (3.70%)	—	—
1	15 (55.56%)	—	—
2	10 (37.04%)	—	—
3	1 (3.70%)	—	—
**MMSE**	27.3 ± 4.9	29.5 ± 1.1	5.35×10^{-2}[a]
**HSCT**	9.4 ± 5.2	12.8 ± 4.5	2.63×10^{-2}[a]
**Methylation of NR4A1**	0.35 ± 0.05	0.26 ± 0.05	1.05×10^{-4}[a]

Notes: Data are expressed as mean ± SD. [a]: Two-sample t test; [b]: Chi-square test.

Abbreviations: TLE, temporal lobe epilepsy; HC, healthy controls; L/B/R, left/bilateral/right epilepsy lateralization; AEDs, Anti-Epileptic Drugs; MMSE, Minimum Mental State Examination; HSCT, Hayling Sentence Complete Test.

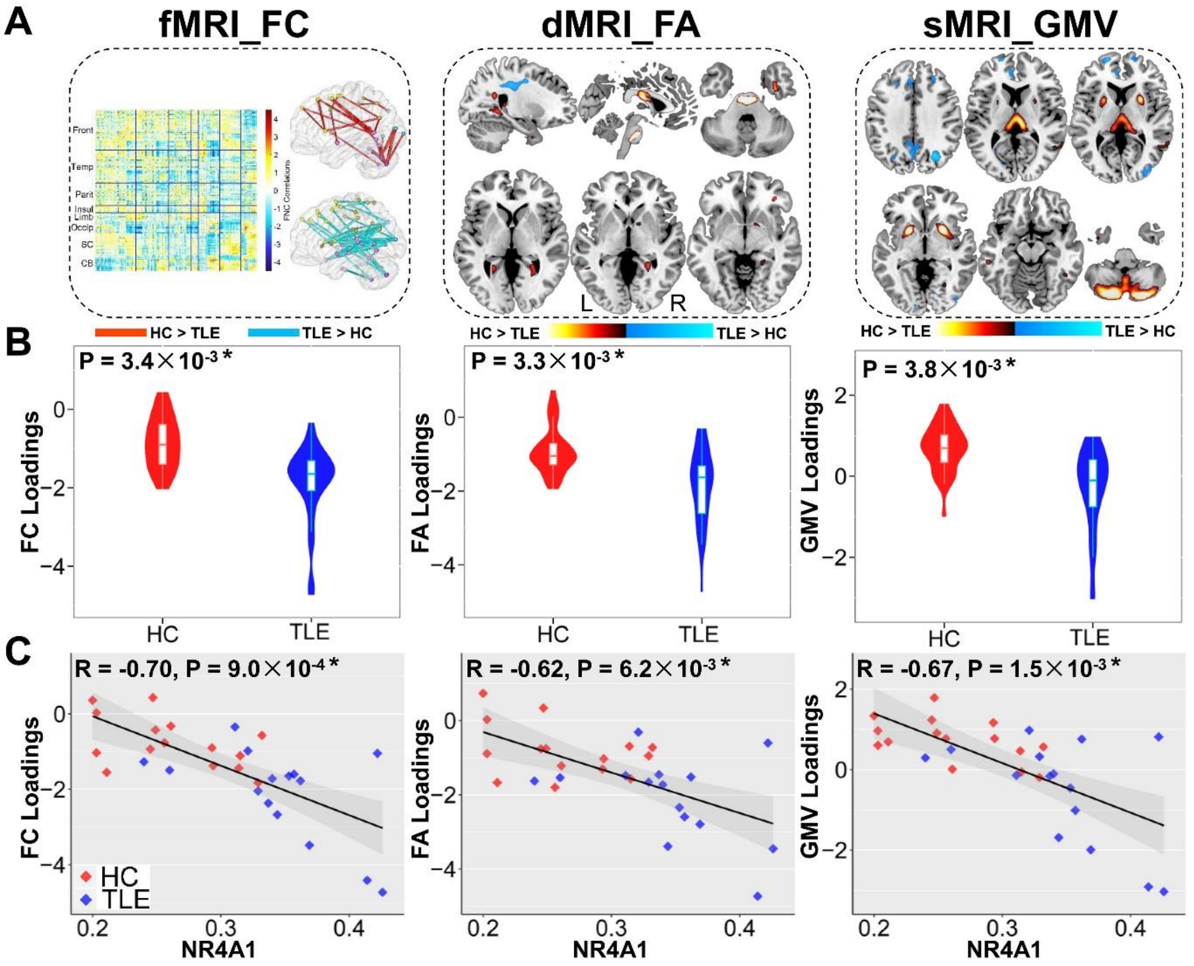

Figure S5. The first joint components associated with methylation levels of NR4A1 in right TLE patients. (A) The spatial maps of FA and GMV were visualized at |Z| > 2, where the positive Z-values (red regions) denotes right TLE < HC and the negative Z-values (blue regions) denotes right TLE > HC. The FC matrix (left) was transformed into z-scores and visualized at |Z| > 3 (right), which displayed positive and negative links separately through the Brant toolbox. (B) Group difference in loading parameters that were adjusted as HC > right TLE on the mean of loadings for each modality. (C) Correlations between loadings of components and methylation levels of NR4A1 (HC: red dots, right TLE: blue dots), in which right TLE patients correspond to higher methylation levels of NR4A1 and lower loadings weights compared to HCs. Note that * means significance passed FDR corrected for multiple comparison and gray regions in c indicate a 95% confidence interval.

Table S3. Demographic and clinical information of left TLE patients and matched HCs.

	Left TLE (N=26)	HC (N=26)	*p* Value
**Age, y**	31.5 ± 11.4	31.2 ± 10.1	0.94[a]
**Male/female**	7/19	13/13	0.15[b]
**Handedness, L/R**	0/26	0/26	—
**Epilepsy lateralization, L/B/R**	26/0/0	—	—
**Age of onset, y**	22.3 ± 11.8	—	—
**Hippocampus Sclerosis: yes/no**	0/26	0/26	—
**Epilepsy duration, y**	9.3 ± 7.3	—	—

**Seizure frequency (proportion)**			
< twice per month	12 (46.15%)	—	—
2-4 times per month	3 (11.54%)	—	—
>4 times per month	11 (42.31%)	—	—
**Number of AEDs（proportion）**		—	—
0	3 (11.54%)	—	—
1	10 (38.46%)	—	—
2	12 (46.15%)	—	—
3	1 (3.85%)	—	—
**MMSE**	27.1 ± 2.9	29.7 ± 1.0	2.91×10^{-4a}
**HSCT**	12.5 ± 4.3	13.1 ± 3.3	0.61^{a}
**Methylation of NR4A1**	0.34 ± 0.07	0.28 ± 0.05	8.50×10^{-3a}

Notes: Data are expressed as mean ± SD. [a]: Two-sample t test; [b]: Chi-square test.

Abbreviations: TLE, temporal lobe epilepsy; HC, healthy controls; L/B/R, left/bilateral/right epilepsy lateralization; AEDs, Anti-Epileptic Drugs; MMSE, Minimum Mental State Examination; HSCT, Hayling Sentence Complete Test.

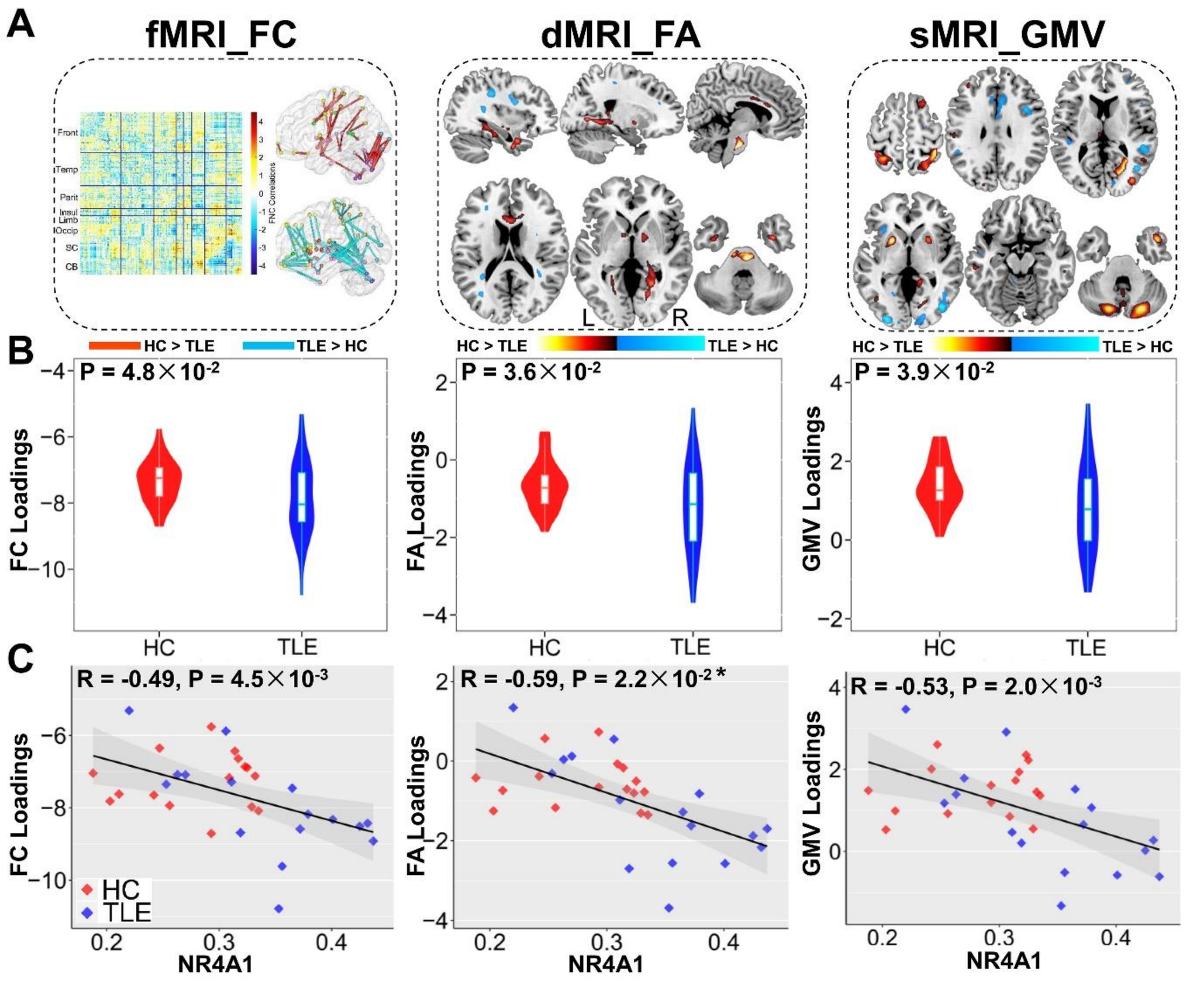

Figure S6. The first joint components associated with methylation levels of NR4A1 in left TLE patients.(A) The spatial maps of FA and GMV were visualized at |Z| > 2, where the positive Z-values (red regions) denotes left TLE < HC and the negative Z-values (blue regions) denotes left TLE > HC. The FC matrix (left) was transformed into z-scores and visualized at |Z| > 3 (right), which displayed positive and negative links separately through the Brant toolbox. (B) Group difference in loading parameters that were adjusted as HC > left TLE on the mean of loadings for each modality. (C) Correlations between loadings of components and methylation levels of NR4A1 (HC: red dots, left

TLE: blue dots), in which left TLE patients correspond to higher methylation levels of NR4A1 and lower loadings weights compared to HCs. Note that * means significance passed FDR corrected for multiple comparison and gray regions in c indicate a 95% confidence interval.

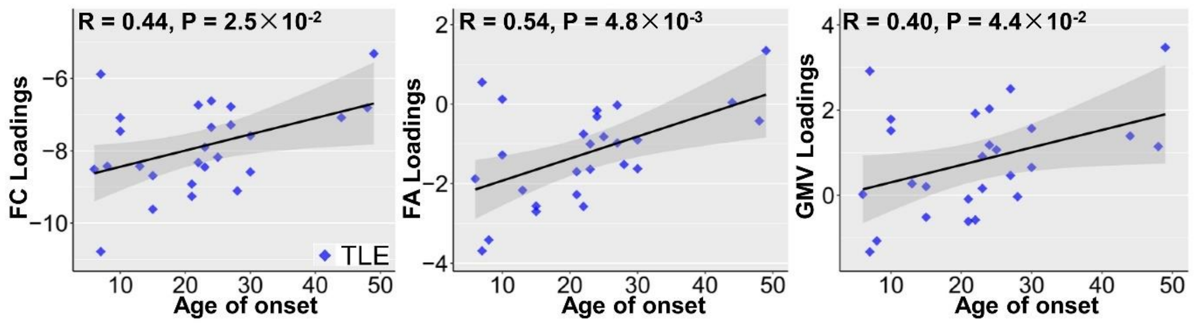

Figure S7. Positive correlations between loadings of joint components and age at onset of left TLE patients. Earlier left TLE onset corresponds to lower loading weights in three modalities, suggesting more serious disability in epilepsy.

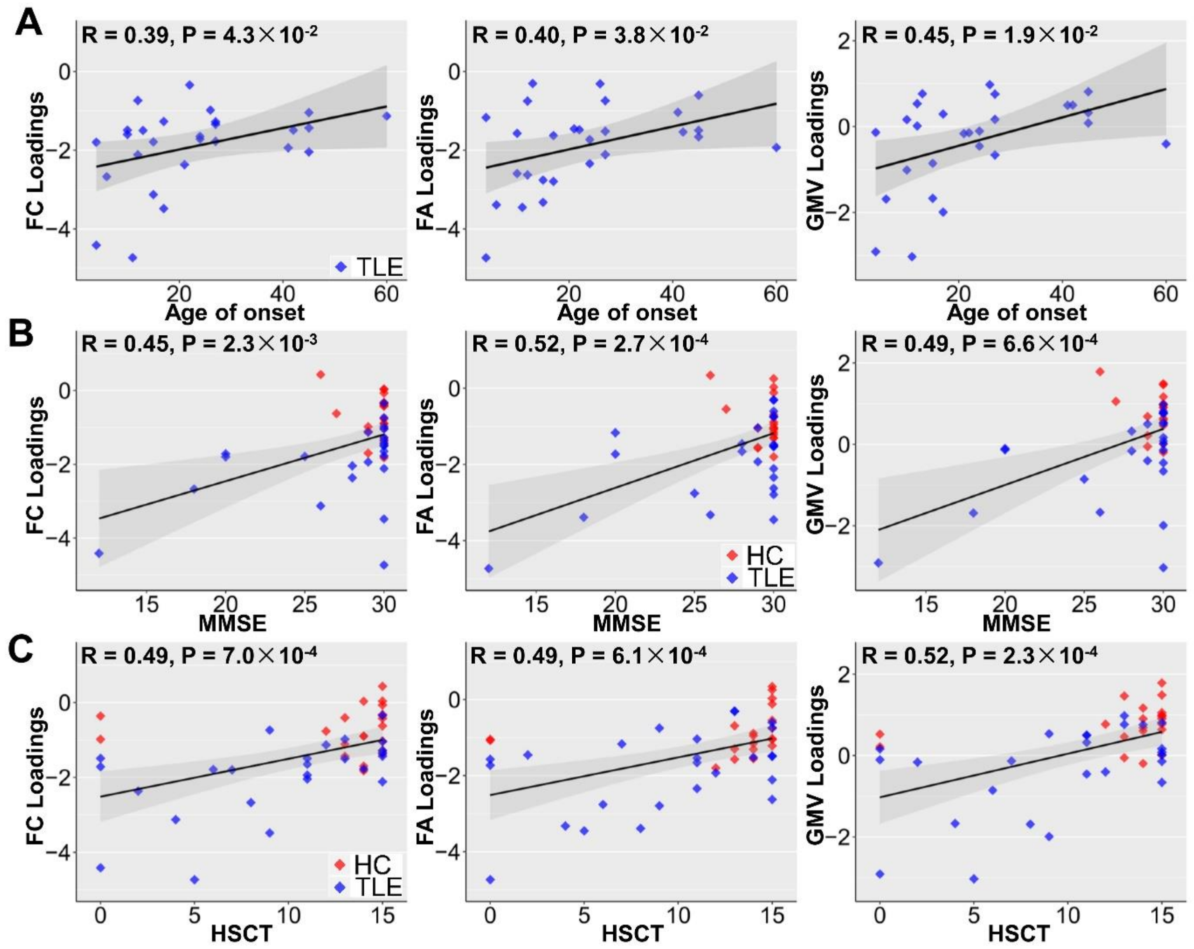

Figure S8. Correlations between loadings of identified components and age of onset (A), MMSE (B), and HSCT scores (C) in right TLE patients. Earlier TLE onset corresponds to lower loading weights in three modalities, suggesting more serious disability in epilepsy. Higher MMSE scores are associated with better cognitive ability and higher HSCT scores correspond to better executive function, which are linked with higher loading weights. Note that gray regions in A, B, and C indicate a 95% confidence interval.

8. Quantitative analysis for the lateralization

In order to give a quantitative analysis for the lateralization, the spatial maps of GMV for right and left thalamus and putamen were extracted respectively through AAL template (3 mm^3). We further performed histogram statistics on voxels with absolute activation value greater than 2 for right and left thalamus and putamen respectively. As shown in Figure S9, the right regions were larger than left regions for right TLE while the left regions were larger than right regions for left TLE regardless of the activation intensity or the number of activated voxels. Thus, the thalamus and putamen showed more impairment in GMV on the side of seizure origin compared with the contralateral side. In addition, it is observed that right TLE showed more GMV reduction in thalamus compared with left TLE.

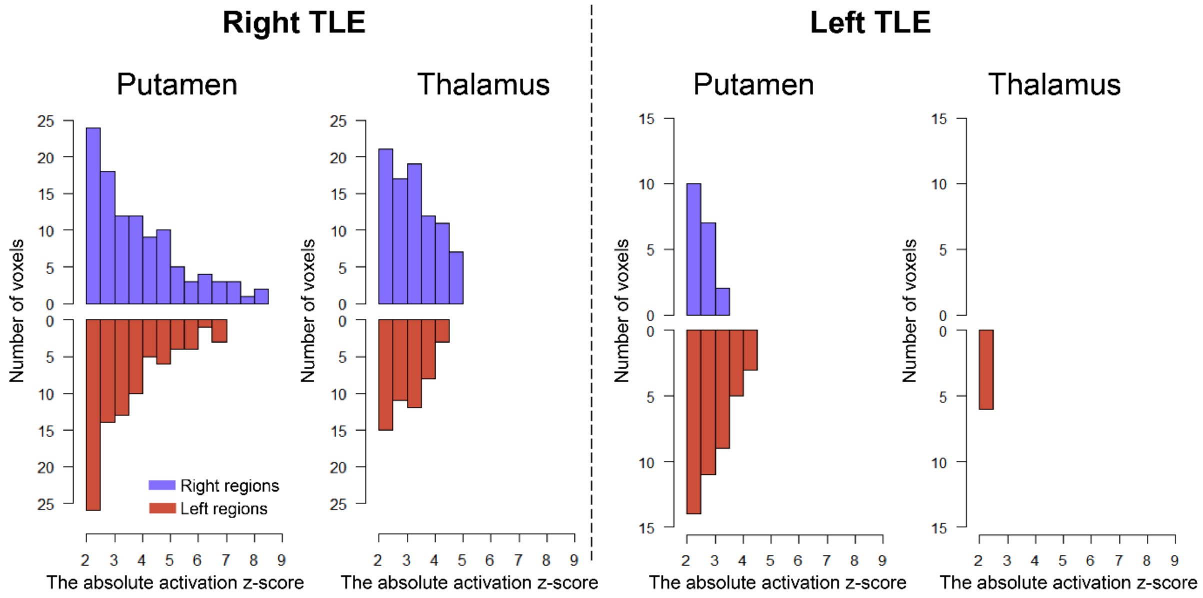

Figure S9. The histogram statistics of grey matter volume in the left and right thalamus and putamen for right TLE and left TLE patients respectively, in which blue bars denotes right regions and red bars denotes left regions for thalamus or putamen.

9. Tables for detailed functional connectivity

Table S4. The detailed functional connectivity thresholded at |Z| > 3 for all TLE.

**Increased Connectivity (region 1 - region 2)**			
MFG_R_7_1 - SFG_L_7_2	IFG_R_6_2 - MFG_R_7_4	CB_Right_IX - ITG_R_7_4	CG_L_7_7 - INS_L_6_6
CB_Left_Crus_II - SFG_L_7_2	IFG_R_6_2 - MFG_R_7_5	FuG_L_3_1 - ITG_L_7_5	CB_Right_Crus_I - CG_L_7_1
CB_Left_Crus_II - SFG_R_7_2	IPL_R_6_2 - IFG_R_6_2	pSTS_R_2_2 - ITG_R_7_5	CB_Left_Crus_II - CG_L_7_1
MFG_L_7_5 - SFG_L_7_3	PoG_L_4_4 - PrG_L_6_4	CB_Right_VI - ITG_R_7_7	CB_Left_Crus_I - CG_R_7_1
IFG_L_6_2 - SFG_R_7_3	STG_L_6_5 - STG_L_6_3	CB_Vermis_VIIb - ITG_R_7_7	CB_Right_Crus_I - CG_R_7_1
IFG_R_6_6 - SFG_R_7_3	ITG_L_7_5 - STG_L_6_4	CB_Vermis_VIIIa - ITG_R_7_7	CB_Left_Crus_II - CG_R_7_1
IFG_R_6_6 - SFG_L_7_6	IPL_L_6_5 - MTG_L_4_1	FuG_R_3_3 - FuG_R_3_1	CB_Right_Crus_II - CG_R_7_1
IFG_R_6_6 - SFG_R_7_6	ITG_L_7_3 - MTG_L_4_2	CB_Left_Crus_I - IPL_L_6_2	CB_Right_Crus_I - CG_R_7_4
IFG_R_6_6 - SFG_L_7_7	IPL_L_6_5 - MTG_L_4_4	CB_Left_Crus_II - IPL_R_6_2	Amyg_R_2_1 - LOcC_L_4_1
IFG_R_6_6 - SFG_R_7_7	CB_Left_IX - ITG_R_7_4	CB_Right_Crus_I - PCun_R_4_4	Amyg_R_2_2 - LOcC_L_4_1
**Decreased Connectivity (region 1 - region 2)**			
CB_Right_IX - SFG_L_7_5	CB_Right_Crus_II - FuG_L_3_3	INS_R_6_4 - INS_L_6_4	BG_L_6_4 - BG_R_6_2
OrG_R_6_3 - OrG_L_6_1	CB_Left_Crus_I - FuG_R_3_3	Tha_R_8_2 - INS_L_6_6	BG_L_6_6 - BG_R_6_2

OrG_R_6_3 - OrG_R_6_1	CB_Left_Crus_II - FuG_R_3_3	LOcC_R_4_3 - MVOcC_L_5_3	Tha_L_8_2 - BG_R_6_2
OrG_L_6_5 - OrG_L_6_3	PhG_R_6_1 - PhG_L_6_1	LOcC_L_4_3 - MVOcC_R_5_3	Tha_L_8_3 - BG_R_6_2
OrG_R_6_5 - OrG_L_6_3	PhG_R_6_5 - PhG_L_6_1	LOcC_L_4_4 - MVOcC_R_5_4	Tha_L_8_5 - BG_R_6_2
OrG_L_6_5 - OrG_R_6_3	CG_L_7_4 - pSTS_L_2_2	CB_Left_Crus_I - LOcC_L_4_1	Tha_L_8_2 - BG_L_6_3
OrG_R_6_5 - OrG_R_6_3	CG_R_7_4 - pSTS_L_2_2	CB_Left_Crus_II - LOcC_L_4_1	Tha_L_8_5 - BG_L_6_5
CG_L_7_1 - STG_R_6_1	CG_L_7_1 - pSTS_R_2_2	CB_Right_Crus_II - LOcC_L_4_1	Tha_L_8_2 - BG_L_6_6
IPL_L_6_2 - STG_L_6_2	CG_R_7_4 - pSTS_R_2_2	CB_Left_Crus_II - LOcC_R_4_1	Tha_L_8_3 - Tha_R_8_2
Tha_R_8_2 - STG_L_6_5	PCun_L_4_2 - SPL_L_5_1	CB_Right_Crus_II - LOcC_R_4_1	CB_Left_VI - CB_Right_V
FuG_L_3_1 - ITG_R_7_3	CG_L_7_6 - SPL_L_5_1	CB_Left_Crus_II - LOcC_L_4_3	CB_Vermis_VI - CB_Right_V
FuG_R_3_1 - ITG_R_7_3	PCun_L_4_2 - SPL_R_5_1	CB_Left_Crus_I - LOcC_L_4_4	CB_Vermis_VI - CB_Left_VI
FuG_R_3_1 - FuG_L_3_1	CG_L_7_6 - SPL_R_5_1	CB_Left_Crus_II - LOcC_L_4_4	CB_Right_VI - CB_Left_VI
PhG_R_6_5 - FuG_L_3_1	PCun_L_4_2 - SPL_R_5_2	CB_Left_Crus_I - LOcC_R_4_4	CB_Right_VI - CB_Vermis_VI
PhG_L_6_1 - FuG_R_3_1	CG_L_7_6 - SPL_R_5_4	CB_Right_Crus_I - LOcC_R_4_4	CB_Left_Crus_I - CB_Right_VI
CB_Left_Crus_I - FuG_L_3_2	CG_L_7_1 - IPL_L_6_6	CB_Left_Crus_II - LOcC_R_4_4	CB_Left_IX - CB_Left_VIIIb
CB_Left_Crus_II - FuG_L_3_2	CG_R_7_1 - IPL_L_6_6	Tha_L_8_2 - Amyg_L_2_1	CB_Left_IX - CB_Right_VIIIb
CB_Left_Crus_I - FuG_R_3_2	CB_Left_IX - IPL_L_6_6	BG_R_6_2 - Amyg_R_2_1	
CB_Left_Crus_II - FuG_R_3_2	CB_Right_IX - IPL_L_6_6	Amyg_R_2_2 - Amyg_L_2_2	
CB_Left_Crus_II - FuG_L_3_3	CG_L_7_6 - IPL_R_6_6	BG_L_6_3 - BG_R_6_2	

Note: the region name was used the abbreviation of the BN atlas (http://atlas.brainnetome.org/download.html).

Table S5. The detailed functional connectivity thresholded at |Z| > 3 for right TLE.

Increased Connectivity (region 1 - region 2)			
CB_Left_Crus_II - SFG_R_7_2	SPL_L_5_4 - PrG_L_6_2	LOcC_L_4_1 - STG_L_6_4	CB_Left_IX - ITG_R_7_1
CB_Left_IX - SFG_R_7_3	PoG_L_4_3 - PrG_L_6_2	LOcC_R_4_1 - STG_L_6_4	CB_Right_IX - ITG_R_7_1
SFG_R_7_5 - SFG_L_7_4	LOcC_L_4_1 - PrG_L_6_5	LOcC_L_4_1 - STG_R_6_4	CB_Left_IX - ITG_R_7_4
SPL_R_5_1 - SFG_L_7_4	LOcC_L_4_2 - PrG_L_6_5	LOcC_R_4_1 - STG_R_6_4	CB_Right_IX - ITG_R_7_4
CB_Right_Crus_I - SFG_L_7_6	LOcC_L_2_2 - PrG_L_6_5	LOcC_L_4_2 - STG_R_6_4	MVOcC_L_5_4 - FuG_L_3_3
CB_Left_Crus_II - SFG_R_7_6	LOcC_L_2_2 - PCL_R_2_2	LOcC_L_2_2 - STG_R_6_4	LOcC_L_4_1 - pSTS_L_2_1
CB_Right_Crus_II - MFG_L_7_2	ITG_L_7_5 - STG_L_6_2	LOcC_L_4_1 - STG_R_6_5	LOcC_R_4_1 - pSTS_L_2_1
CB_Right_Crus_I - MFG_L_7_5	LOcC_L_4_1 - STG_L_6_2	LOcC_L_4_2 - STG_R_6_5	LOcC_L_2_2 - IPL_R_6_6
CB_Right_Crus_I - MFG_L_7_6	LOcC_R_4_1 - STG_L_6_2	LOcC_L_2_2 - STG_R_6_5	LOcC_L_4_1 - PoG_R_4_1
PrG_R_6_5 - IFG_L_6_6	LOcC_L_4_2 - STG_L_6_2	LOcC_L_4_1 - STG_L_6_6	LOcC_L_4_1 - PoG_L_4_4
IPL_L_6_3 - IFG_L_6_6	LOcC_L_2_2 - STG_L_6_2	LOcC_L_4_2 - STG_L_6_6	LOcC_L_4_1 - INS_R_6_4
IPL_L_6_6 - IFG_L_6_6	MTG_R_4_3 - STG_R_6_2	LOcC_L_2_2 - STG_L_6_6	LOcC_L_2_2 - INS_R_6_4
IPL_R_6_6 - IFG_L_6_6	LOcC_L_4_2 - STG_R_6_2	LOcC_L_4_2 - STG_R_6_6	LOcC_L_4_1 - INS_R_6_5
INS_R_6_3 - IFG_L_6_6	LOcC_L_4_1 - STG_L_6_3	IPL_L_6_6 - MTG_L_4_3	LOcC_L_2_2 - INS_L_6_6
INS_L_6_6 - IFG_L_6_6	LOcC_L_4_2 - STG_L_6_3	IPL_L_6_5 - MTG_L_4_4	LOcC_L_2_2 - INS_R_6_6
INS_R_6_6 - IFG_L_6_6	LOcC_L_2_2 - STG_R_6_3	PCun_L_4_4 - MTG_L_4_4	
**Decreased Connectivity (region 1 - region 2)**			
IFG_L_6_6 - SFG_L_7_2	CB_Left_Crus_II - FuG_R_3_2	CB_Right_Crus_I - FuG_R_3_3	CB_Right_Crus_II - LOcC_L_4_1
MTG_L_4_3 - SFG_L_7_2	CB_Left_Crus_II - FuG_R_3_2	IPL_L_6_2 - SPL_R_5_3	CB_Left_Crus_I - LOcC_R_4_1
pSTS_L_2_2 - SFG_L_7_2	CB_Left_Crus_II - FuG_R_3_2	IPL_R_6_3 - IPL_L_6_2	CB_Right_Crus_I - LOcC_R_4_1
IFG_L_6_6 - SFG_R_7_2	CB_Left_Crus_II - FuG_R_3_2	LOcC_L_2_2 - IPL_L_6_2	CB_Left_Crus_II - LOcC_R_4_1
IFG_L_6_6 - SFG_R_7_3	CB_Left_Crus_II - FuG_R_3_2	INS_R_6_4 - INS_L_6_4	CB_Right_Crus_I - LOcC_R_4_2
MTG_R_4_3 - SFG_R_7_3	CB_Left_Crus_II - FuG_R_3_2	CB_Vermis_VIIIb - INS_R_6_6	CB_Left_Crus_II - LOcC_L_4_3
CG_R_7_5 - SFG_L_7_5	CB_Left_Crus_II - FuG_R_3_2	CB_Left_Crus_II - MVOcC_R_5_4	CB_Right_Crus_I - CB_Right_VI

MTG_L_4_3 - MFG_L_7_6	CB_Left_Crus_II - FuG_R_3_2	CB_Left_Crus_I - LOcC_L_4_1	CB_Left_Crus_II - CB_Right_VI
MTG_R_4_3 - MFG_L_7_6	CB_Left_Crus_II - FuG_R_3_2	CB_Right_Crus_I - LOcC_L_4_1	
ITG_L_7_5 - MFG_L_7_6	CB_Left_Crus_II - FuG_R_3_2	CB_Left_Crus_II - LOcC_L_4_1	

Note: the region name was used the abbreviation of the BN atlas (http://atlas.brainnetome.org/download.html).

Table S6. The detailed functional connectivity thresholded at |Z| > 3 for left TLE.

**Increased Connectivity (region 1 - region 2)**			
INS_L_6_6 - SFG_L_7_1	BG_R_6_3 - OrG_R_6_4	LOcC_R_4_1 - PoG_L_4_3	CB_Left_Crus_I - Tha_L_8_3
OrG_R_6_6 - SFG_L_7_3	PoG_L_4_3 - PrG_L_6_3	CB_Left_Crus_I - CG_R_7_1	CB_Left_Crus_I - Tha_R_8_3
INS_L_6_2 - SFG_L_7_3	PoG_L_4_4 - PrG_L_6_4	CB_Vermis_VIIIa - CG_R_7_1	CB_Left_Crus_I - Tha_L_8_4
IFG_R_6_6 - SFG_R_7_3	PCL_R_2_2 - PrG_R_6_4	CB_Right_Crus_I - CG_R_7_4	CB_Left_Crus_I - Tha_R_8_4
OrG_R_6_6 - SFG_R_7_3	STG_L_6_5 - STG_L_6_3	MVOcC_L_5_5 - MVOcC_L_5_1	CB_Left_Crus_I - Tha_L_8_5
INS_L_6_3 - SFG_L_7_6	IPL_R_6_5 - MTG_R_4_1	MVOcC_L_5_5 - MVOcC_L_5_4	CB_Left_Crus_I - Tha_R_8_5
IFG_R_6_2 - MFG_R_7_5	INS_L_6_2 - MTG_L_4_2	MVOcC_L_5_5 - MVOcC_R_5_4	CB_Left_Crus_I - Tha_R_8_6
INS_R_6_3 - MFG_R_7_5	Amyg_L_2_1 - MTG_L_4_2	LOcC_L_2_2 - LOcC_R_4_1	CB_Right_Crus_II - Tha_R_8_6
BG_R_6_2 - MFG_R_7_6	SPL_R_5_3 - MTG_R_4_3	CB_Left_Crus_I - BG_L_6_2	CB_Left_Crus_I - Tha_L_8_7
BG_R_6_6 - MFG_R_7_6	IPL_L_6_5 - MTG_L_4_4	CB_Left_Crus_I - BG_R_6_2	CB_Right_Crus_II - Tha_L_8_7
IFG_L_6_6 - IFG_L_6_5	IPL_R_6_5 - MTG_R_4_4	CB_Left_Crus_I - BG_L_6_5	CB_Left_Crus_I - Tha_R_8_7
OrG_R_6_4 - OrG_R_6_1	CB_Right_VI - ITG_R_7_7	Tha_R_8_7 - BG_R_6_5	CB_Left_Crus_II - Tha_R_8_7
OrG_R_6_6 - OrG_L_6_4	PoG_L_4_2 - FuG_L_3_2	CB_Left_Crus_I - BG_R_6_5	CB_Right_Crus_II - Tha_R_8_7
OrG_L_6_5 - OrG_R_6_4	PoG_L_4_2 - FuG_R_3_2	CB_Left_Crus_I - Tha_L_8_1	CB_Left_Crus_I - Tha_L_8_8
OrG_R_6_5 - OrG_R_6_4	PoG_L_4_3 - FuG_R_3_2	CB_Left_Crus_II - Tha_L_8_1	CB_Left_Crus_II - Tha_L_8_8
ITG_R_7_3 - OrG_R_6_4	PoG_L_4_2 - FuG_L_3_3	Tha_L_8_7 - Tha_R_8_1	CB_Right_Crus_II - Tha_L_8_8
CG_R_7_2 - OrG_R_6_4	PoG_L_4_3 - SPL_R_5_4	CB_Left_Crus_I - Tha_R_8_1	CB_Left_Crus_I - Tha_R_8_8
CG_L_7_7 - OrG_R_6_4	IPL_L_6_3 - SPL_L_5_5	CB_Right_Crus_II - Tha_R_8_1	CB_Right_Crus_II - Tha_R_8_8
BG_L_6_1 - OrG_R_6_4	MVOcC_R_5_4 - PoG_L_4_2	CB_Left_Crus_I - Tha_L_8_2	CB_Vermis_VIIIa - CB_Left_Crus_II
BG_R_6_1 - OrG_R_6_4	PoG_L_4_4 - PoG_L_4_3	CB_Left_Crus_II - Tha_L_8_2	
BG_L_6_3 - OrG_R_6_4	LOcC_L_4_1 - PoG_L_4_3	CB_Left_Crus_I - Tha_R_8_2	
**Decreased Connectivity (region 1 - region 2)**			
Tha_L_8_8 - SFG_L_7_5	CB_Vermis_VI - FuG_L_3_2	CB_Vermis_Crus_II - MVOcC_R_5_1	CB_Right_Crus_II - LOcC_R_4_4
OrG_R_6_4 - MFG_R_7_7	CB_Left_Crus_I - FuG_R_3_2	CB_Left_Crus_I - LOcC_L_4_1	BG_R_6_2 - Amyg_L_2_1
OrG_L_6_5 - OrG_L_6_3	CB_Right_Crus_II - FuG_L_3_3	CB_Left_Crus_II - LOcC_L_4_1	Tha_L_8_2 - Amyg_L_2_1
PrG_R_6_6 - PrG_L_6_1	PCun_L_4_3 - pSTS_R_2_2	CB_Right_Crus_II - LOcC_L_4_1	BG_R_6_2 - Amyg_R_2_1
PrG_R_6_6 - PrG_R_6_1	PCun_R_4_3 - pSTS_R_2_2	CB_Left_Crus_I - LOcC_R_4_1	Tha_R_8_2 - Amyg_R_2_1
Tha_R_8_4 - PrG_L_6_4	PCun_R_4_4 - pSTS_R_2_2	CB_Left_Crus_II - LOcC_R_4_1	CB_Right_IX - Amyg_R_2_1
BG_R_6_6 - PrG_L_6_5	CG_R_7_4 - pSTS_R_2_2	CB_Right_Crus_II - LOcC_R_4_1	BG_R_6_2 - Amyg_L_2_2
CG_L_7_2 - PCL_L_2_2	CG_L_7_6 - SPL_L_5_1	CB_Vermis_VI - LOcC_L_4_3	BG_R_6_2 - Amyg_R_2_2
CG_L_7_2 - PCL_R_2_2	Tha_L_8_1 - PoG_L_4_3	CB_Left_Crus_II - LOcC_L_4_3	BG_L_6_3 - BG_R_6_2
MTG_R_4_1 - STG_L_6_2	Tha_R_8_7 - PoG_L_4_3	CB_Vermis_VI - LOcC_L_4_4	Tha_L_8_2 - BG_L_6_3
IPL_L_6_2 - STG_L_6_2	CG_L_7_2 - PoG_L_4_4	CB_Left_Crus_I - LOcC_L_4_4	CB_Vermis_VIIIb - CB_Left_VIIIa
MTG_R_4_1 - STG_R_6_2	CG_L_7_2 - PoG_R_4_4	CB_Left_Crus_II - LOcC_L_4_4	CB_Vermis_VIIIb - CB_Left_VIIIb
PoG_L_4_1 - STG_L_6_3	BG_R_6_6 - INS_R_6_6	CB_Right_Crus_II - LOcC_L_4_4	CB_Left_IX - CB_Left_VIIIb
CG_L_7_4 - MTG_R_4_3	CB_Vermis_VI - MVOcC_L_5_1	CB_Left_Crus_I - LOcC_R_4_4	
CG_R_7_4 - MTG_R_4_3	CB_Left_Crus_II - MVOcC_L_5_1	CB_Right_Crus_I - LOcC_R_4_4	
FuG_R_3_1 - FuG_L_3_1	CB_Vermis_VI - MVOcC_R_5_1	CB_Left_Crus_II - LOcC_R_4_4	

Note: the region name was used the abbreviation of the BN atlas (http://atlas.brainnetome.org/download.html).

10. References

Du, Y., Fryer, S.L., Fu, Z., Lin, D., Sui, J., Chen, J., Damaraju, E., Mennigen, E., Stuart, B., and Loewy, R.L. (2018). Dynamic functional connectivity impairments in early schizophrenia and clinical high-risk for psychosis. *Neuroimage* 180**,** 632-645.

Xu, K., Liu, Y., Zhan, Y., Ren, J., and Jiang, T. (2018). BRANT: A Versatile and Extendable Resting-State fMRI Toolkit. *Frontiers in neuroinformatics* 12.
